# A Case of Gas Gangrene Exhibiting Unusual Proofs of a Blood Infection

**Published:** 1917-12

**Authors:** 


***A Case of Gas Gangrene Exhibiting Unusual Proofs of a Blood Infection.*** By G. T. MULLALLY and J. W. MCNEE, Capts. R. A. M. C. Abs. from the *British Medical Journal*, April 1, 1916.

The authors report a single case which they wish to record as proof of blood infection by gas-forming organisms. They state that it is the only one of its kind that they have ever observed.

A patient wounded on March 18, 1915 developed gas gangrene on March 21 at the site of the wound (right arm with fractured humerous). On March 19 an anti-tetanic injection was made below the left nipple. On March 20 a hypodermic injection of pituitrin was made in the left fore-arm. On March 20 he complained of pain in the region of the serum injection, and on the following day a patch of gas gangrene appeared there. On March 22 the site of the pituitrin injection also showed an area of gas gangrene. All of the infected areas were excised. That of the chest wall near the point of the anti-tetanic injection involved the skin and subcutaneous tissue only. The structures below the deep fascia were not invaded.

Bacterial examination of all three regions disclosed the presence of bacilli of the malignant edema type, showing central spores, and accompanied with a variety of other organisms.

Cultures were made from all three regions in various media. (1) Shake culture in large tubes of glucose agar. (2) Broth cultures made partly anaerobic. The malignant edema organism was obtained by both methods from all the regions, and from the blood as well. In all the glucose agar tubes there was a marked production of gas.

